# Area efficient folded undecimator based ECG detector

**DOI:** 10.1038/s41598-021-82231-2

**Published:** 2021-02-12

**Authors:** A. Uma, P. Kalpana

**Affiliations:** PSG College of Technology, Coimbatore, Tamil Nadu India

**Keywords:** Health care, Medical research

## Abstract

This paper presents an area-efficient folded wavelet filter-based Electrocardiogram (ECG) detector for cardiac pacemakers. The modified folded undecimator based detector consists of Wavelet Filter Bank, QRS complex detector with Generalized Likelihood Ratio Test (GLRT) block and noise detector. A high-level transformation technique such as folding transformation and Cutset retiming are applied to the GLRT block in order to reduce the silicon area. Folding is a high-level transformation applied at the architectural level to enhance the performance of DSP architectures. It reduces the number of adders, multipliers and delay elements in the architecture. The Cutset retiming reduces clock period of the architecture by changing position of delay elements in the critical path. The folding transformation and cutset retiming implement the functional blocks of the GLRT circuit with minimum hardware. The modified folded ECG detector is tested for short term and long-term MIT-BIH databases. The results show that the modified folded undecimator detector has hardware savings and achieves sensitivity of 99.95%, positive prediction of 99.97% and Detection Error Rate (DER) of 0.061. The folded GLRT block architecture is synthesized with FPGA Zed board XC7Z010CLG484-1. Results show that the device utilization and power consumption are lesser than the conventional GLRT structure.

## Introduction

Due to advances in Integrated Circuit (IC) technology, there is a high demand for low-cost implantable devices and body monitoring systems. The implantable medical devices are used for treating health-related problems as well as continuous monitoring^1^. Over the years, the development of these implantable devices gained importance due to advances in microelectronics and signal processing techniques^2^.ECG is an important tool for health professionals in diagnosing cardiac-related problems. An ECG monitoring system consists of a recording and analysing system. During signal acquisition, noises from different sources interfere and affect the quality of signals. Analysis system is used to extract the important features of the acquired signal^3^. The current trend in the ECG detectors makes it more attractive for pacemaker which generate electrical impulse and produce a normal heartbeat on-demand basis. The two main components of pacemakers are Integrated Circuit and battery. The integrated circuits of pacemaker consist of ECG detector, peak counter and pulse generator^4^. The pacemakers should be implanted with less hardware so that the area can be reduced. The rapid growth of biomedical CMOS IC design provides area-efficient ECG detection architectures that have low power consumption^4^. The key features of these architectures implanted in pacemakers provide accuracy in detection also.

In the previous decades, many different types of research were carried out and different methods of QRS detection have been proposed. With the increase of modern technology and more advances in computers, many new approaches in ECG monitoring and diagnosis were developed. An efficient fractional order-based lattice wave digital filter^5^ is realized with a minimum number of multipliers. This lattice filter performs R-peak detection using slope information, dynamic compression, and cross-correlation. A wavelet transform based approach is used for modelling and classification of congestive heart failures^6^. This method uses the LZMA compression algorithm and peak detection with higher sensitivity and predictivity. A biorthogonal wavelet 3.1 based ECG detector and Run Length Encoding scheme^3^ are used for ECG monitoring system. The biorthogonal wavelet-based structure uses Wi-Fi-based transmission and linear phase array structure with higher SNR. Multi-stage adaptive peak detection^7^ is developed using Fast Fourier Transform. A FFT based signal denoising scheme is implemented and heart rate is calculated for different databases. The frequency-domain algorithms use different transforms which include wavelet and Hilbert^8^. A add and shift multiplier is replaced with a booth multiplier^9^ to improve the ECG performance and various on-chip approaches for cardiac pacemakers are studied.

A Biorthogonal wavelet transform based R-peak and data compression scheme^10^ is designed for cardiac pacemakers. This wavelet-based structure uses demand-based filter bank architecture which uses a cascade of three LPF and one HPF. The proposed structure is hardware efficient and consumes low power. The transform-based algorithms have higher detection capability and robustness against noise. A moving average-based method which is combined with wavelet denoising is proposed. The Discrete Wavelet Transform (DWT) is implemented using a Recursive Pyramid algorithm because it requires smaller memory storage than DWT^11^. The main advantage of this method is that it requires few adders and multipliers. The filter bank method consists of a set of analysis and synthesis filters. The function of the analysis filter is to decompose the incoming ECG signal to sub-bands. The synthesis filters are used to reconstruct the original input signal^12^^.^. The filter bank method allows both the time and frequency-based analysis for ECG signals. ECG detection algorithms focus on realization structures to achieve a high detection rate. Hence, a trade-off occurs between circuit complexity and accuracy. A hardware complexity produces more power consumption and area. A R-peak detection using Principle Component Analysis (PCA)^13^ is studied.

Folding transformation is a VLSI signal processing technique used in DSP applications for minimizing the number of functional blocks in architecture. The application of high-level transformations^14^ in DSP blocks alters the structure while maintains the same functionality. The implementation of a silicon chip with minimal hardware is important. This folding algorithm is mapped to the architectural level of ECG detectors and reduces the circuit complexity. This paper implements a Folding transformation and cutset retiming techniques^14,15^ in the (GLRT) block of WFB.

## ECG detection methods

**Figure 1:**
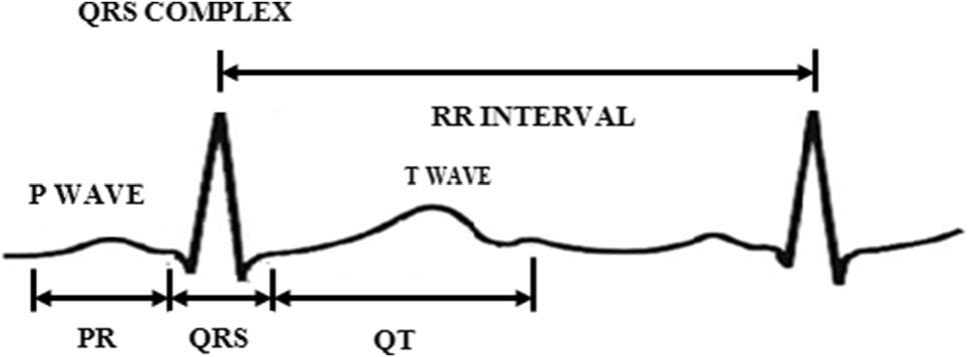
QRS complex waveform.

The ECG waveform has one cardiac cycle which consists of three sections such as P-wave, QRS-complex (Q, R and S wave) and T-wave^16^. The ECG signal is considered as a repeated sequence of the three sections as shown in Fig. 1. During the continuous monitoring of ECG signals, the unwanted noise may interfere and make the detection more complex. The frequency range of the ECG signal is 0.1-250 Hz and frequency components of the QRS waveform lie between 5 and 22 Hz^8^.

### Conventional undecimator based ECG detector

**Figure 2:**
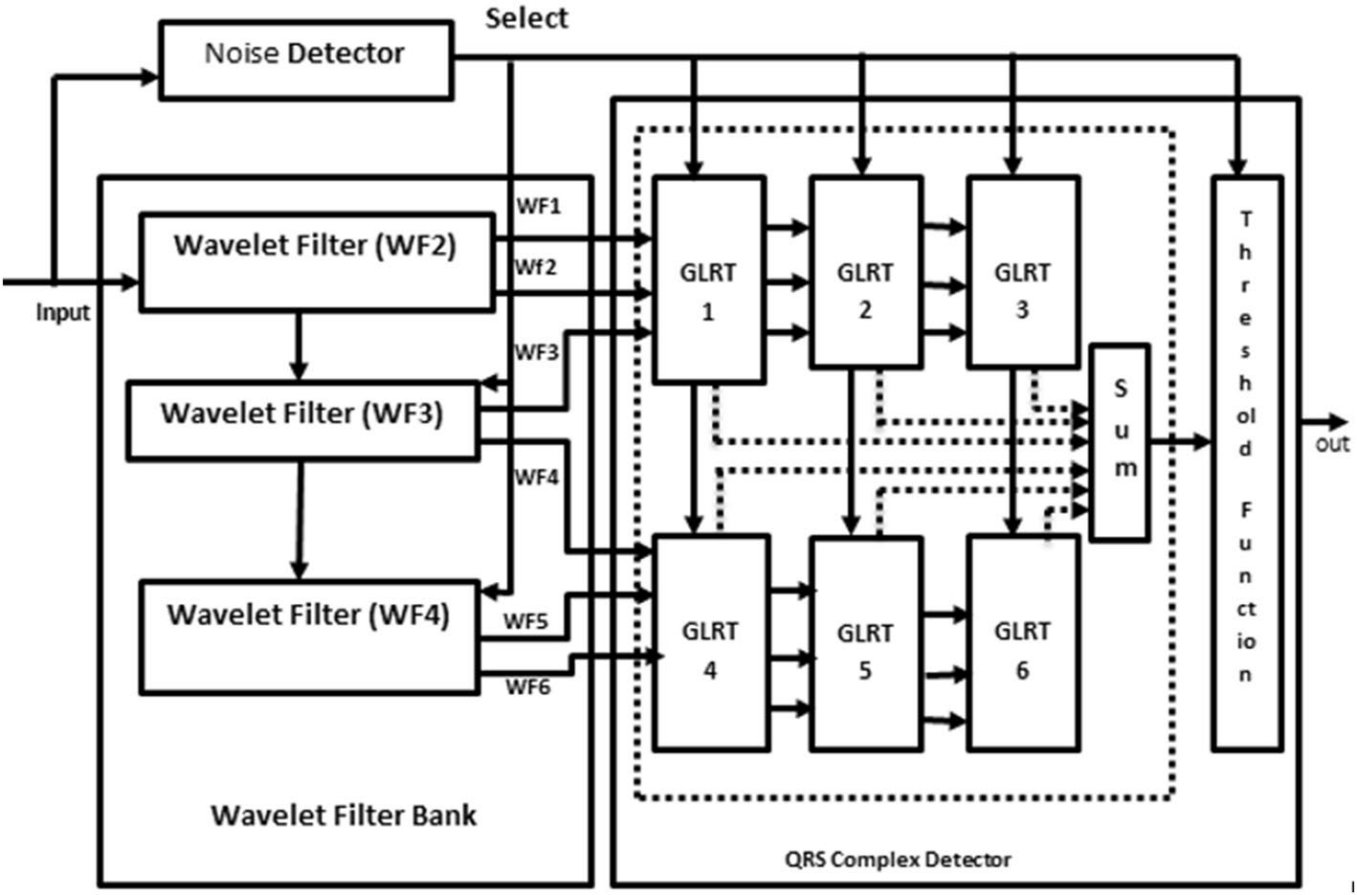
Block diagram of conventional undecimator based ECG detector^4^.

The conventional undecimator based ECG detector^4^ consists of Wavelet Filter Banks (WFBs), a noise detector with zero-crossing points, GLRT and a threshold function block as shown in Fig. 2. The wavelet filter bank produces a monophasic and biphasic outputs. Monophasic waves are the pulse of energy in one direction and biphasic waves are out of phase in two directions. These outputs of WFB are given to the GLRT block as inputs. The noise detector operates the detector in dual mode. The detector uses a select signal applied for QRS detector and WFB. The threshold function compares the output of the GLRT block with a noise detector signal and determines whether it is a cardiac or noise signal.

**Figure 3:**
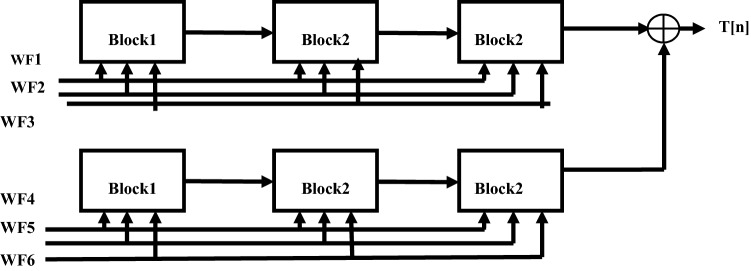
Block diagram of GLRT.

Wavelet Transform (WT) is a powerful tool used for analysing non-stationary signals. In an ECG acquisition system, WT decomposes the incoming signals to multiple components and then it is easily reconstructed to original form without any loss. Both time and frequency information of a signal is extracted using WT. Wavelet has window size which is varied based on the application and suitability of many applications. Hence, wavelet is used to manipulate and extract useful parameters from the ECG signal. For proper diagnosis, these parameters are to be extracted carefully. Three levels of decomposition are selected with scale q = 2, 3, 4. Due to low computational complexity, a dyadic wavelet transform^17^ is used to implement the filter bank which consists of low pass and high pass filters to realize the biphasic and monophasic waves. The efficient realization of both LPF and HPF are performed by sharing common multipliers and delay elements (Fig. 3).

**Figure 4:**
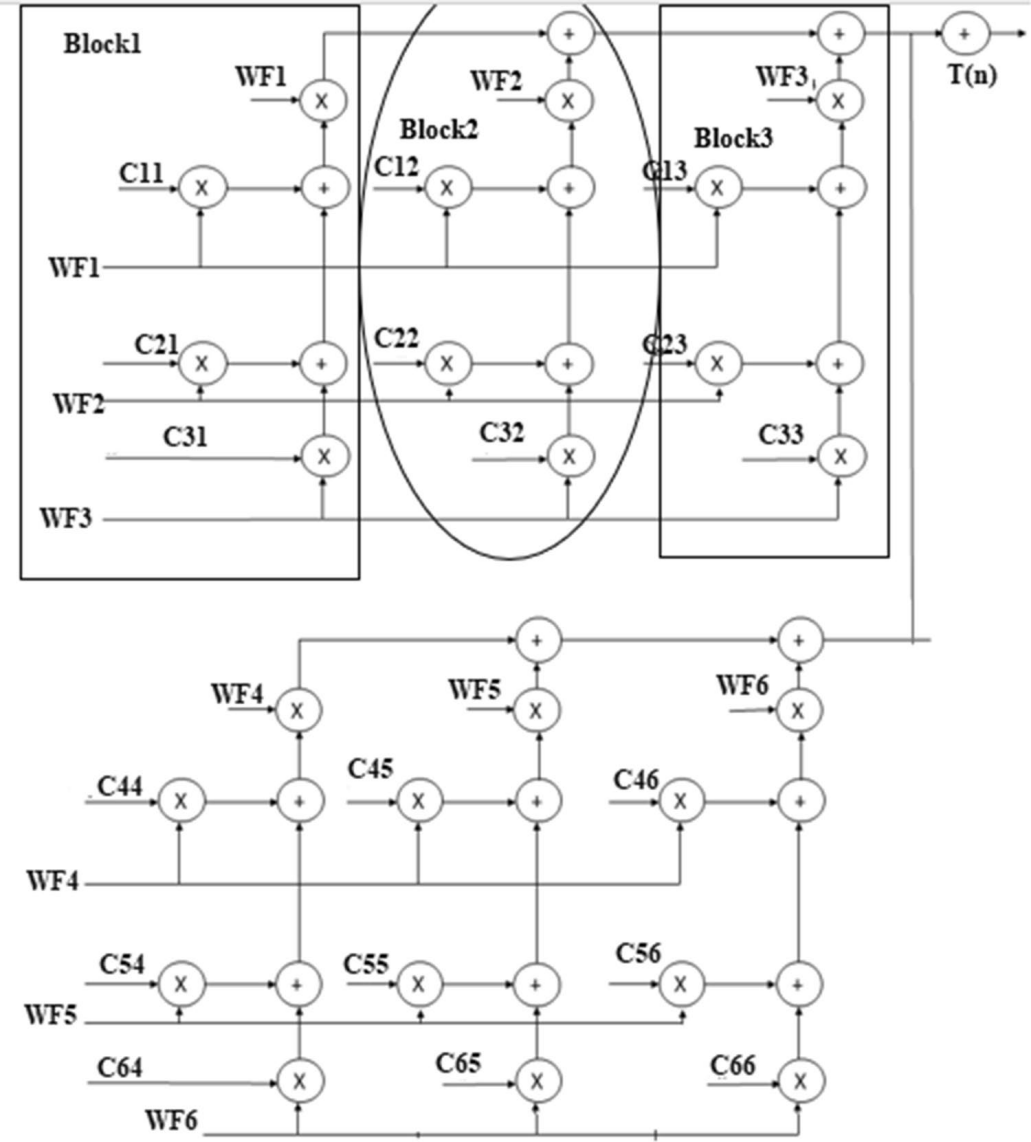
GLRT structure with blocks^4^.

The GLRT evaluates the heart-beat rate of the decomposed WFB outputs with the threshold function and detects the presence of the QRS complex. The structure uses maximum likelihood manipulation with hardware components that include delays, multipliers, and adders. To reduce the hardware requirement a numerical strength reduction technique is adopted in the GLRT block. This method replaces multipliers with the shift-add operation. The GLRT structure with three blocks is shown in Fig. 4. The undecimator based wavelet filter bank outputs, namely WF1 to WF6 are given as input to the GLRT block along with constants C_11_, C_12_ ….C_66_. The GLRT block has two identical stages as shown in Fig. 4

The stage1 has three blocks and similar identical blocks are there in stage2. The structure of stage1 is replicated in stage2 with inputs WF4, WF5, WF6 and coefficients C_44_, C_54_, C_64_^4^. Similarly, block2 and block3 are identical with the same number of 3 adders and 4 multipliers. Each block gets input from the previous blocks with different coefficients. For example, block2 of stage 1 gets input from block1 with coefficients C_12_, C_22_, C_32_ and block3 gets inputs from block2 with coefficients C_13_, C_23_, C_33_. The output of filter bank X ^T^(n) H is multiplied by (H^T^H) ^-1^ which has coefficients C_11_, C_12_ ….C_66_. The hardware is optimized by substituting the constant values in (H^T^ H)^-1^ instead of real values^4^. The overall conventional GLRT diagram with different blocks is presented in Fig. 3. The output of GLRT is expressed as given in Eq. (1)

1 $$T(n) = X^{T} (n)H(H^{T} H)^{ - 1} H^{T} X(n)$$

### Modified undecimator based ECG detector

The modified undecimator based ECG detector consists of Wavelet Filter Banks (WFBs), noise detector and Folded Generalized Likelihood Ratio Test (GLRT).

### Wavelet filer bank (WFB)

The wavelet decomposer used in this paper consists of dyadic WT based filter bank^17^. A careful selection of decomposition levels result in efficient peak detection. Three levels of decomposition are selected with scale q = 2, 3, 4. The transfer function of biphasic function h_1,b_ is expressed as (2) where ‘b’ represents biphasic and ‘m’ represents monophasic (Eq. 6)

2 $$h_{1,b} (n) = g_{b} (n)$$

3 $$h_{2,b} (n) = f(n)*g_{b} (2n)$$

4 $$h_{3} ,_{b} (n) = f(n)*f(2n)*g_{b} (4n)$$

5 $$h_{q,b} (n) = f(n)*...*f(2^{q - 2} n)*g_{b} (2^{q - 1} n)$$

6 $$h_{{q,_{m} }} (n) = f(n)*....*f(2^{q - 2} n)*g_{m} (2^{q - 1} n)$$

The scale values define the resolution in time and frequency plane. The monophasic filter bank can be designed by reusing g_b_(n).The filter functions f(n) and g_b_(n)^4^ are expressed as matrices in (Eq. 7)

7 $$f\left( n \right) = \left[ {1 3 3 1} \right] , g_{b } \left( n \right) = \left[ { - 1 1} \right]$$

To make a power-efficient QRS detection^4^, the function of LPF filter is expressed as H(z) in (Eq. 8) and HPF filter as G(z) in (Eq. 9).

8 $$H(z) = 1 + 3z^{ - 1} + 3z^{ - 2} + z^{ - 3}$$

9 $$G(z) = 1 - z^{ - 1}$$

**Figure 5:**
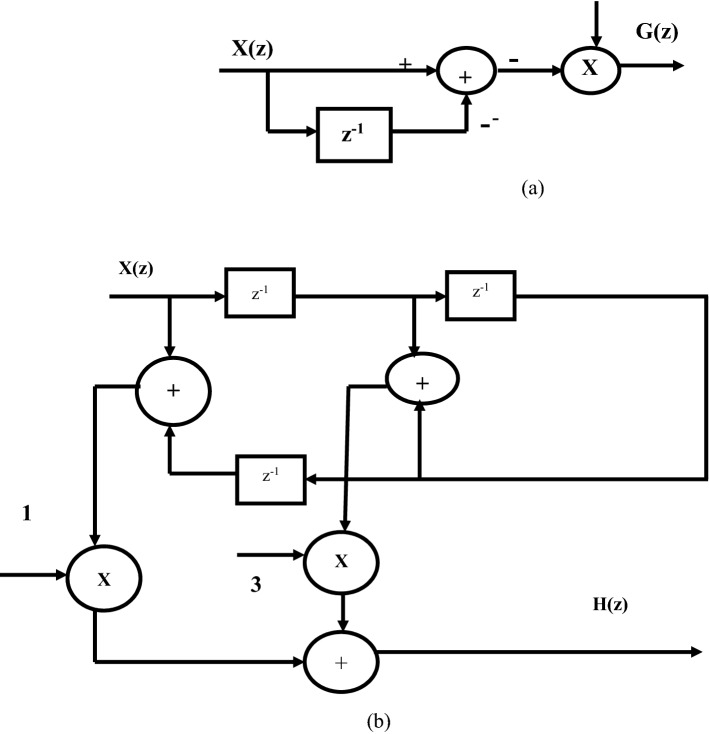
Linear phase realization of (**a**) HPF and (**b**) LPF.

The dyadic wavelet structure uses a pair of undecimator based LPF and HPF. The dyadic based wavelet filter bank is a combination of the asymmetric and symmetric filter. The asymmetric filter approximates biphasic wave and the symmetric filter is used for monophasic wave. At each step of the filter bank, the signal is high-pass and low-pass filtered to extract information from the signal. The high-frequency components of the signal are extracted at the first level of decomposition and low-frequency components are extracted at the other stages. The LPF and HPF filter realizations of the conventional detector have hardware complexity. It uses more delay elements and affects the speed. The hardware requirements and complexity of the architecture highly depend on the selection of filter realization. Based on the realization, the required number of adders, delays, and multipliers varies. Therefore, efficient realization of both the filters can be achieved by using linear phase structure^18^ as shown in Fig. 5. The Linear phase realization structure passes entire frequency components of input ECG signal with equal delay and also group delay is constant. So, linear phase structure is adopted in real-time applications where distortions are more^18^.

The output of WFB is calculated using (Eq. 10) and given as input to GLRT block.

The equations of the output of WFB are expressed as

10 $$w(n) = X^{T} (n)H$$

where $$X(n) = [x(n)...x(n + N - 1)]^{T}$$, $$H = [\tilde{H}_{b} \tilde{H}_{m} ]$$, $$\widetilde{{ H_{b} }} = \left[ {\tilde{h}_{2,b} \tilde{h}_{3,b} \tilde{h}_{4,b} } \right]$$ , $$\widetilde{{H_{m} }} = \left[ {\tilde{h}_{2,m} \tilde{h}_{3,m} \tilde{h}_{4,m} } \right]$$.

#### Folded GLRT

A conventional GLRT block^4^ is folded using folding transformation technique and it results in area reduction. The functional blocks of a folded structure are adders, multipliers, delay elements, and switches. The N operations performed in the original system are replaced as a time-multiplexed single operation in the folded structure. The flexibility in a folded structure is that it can be reused to perform the same N operations in N time units. The drawback of folding transformation is the usage of more delay elements for temporary data storage. But the delay elements required are reduced using the Register minimization technique significantly^20,21^. The optimized folded structure reduces the area and power.

In folding transformation, the trade-off occurs between the area and speed. Due to the delay elements (Registers), the speed gets affected. This work uses the Register minimization technique to improve the speed by optimizing the number of delays. Folding transformation technique is explained by considering a DSP program with adders. A DSP program uses two adders to add input samples a_1_(n), a_2_(n) and a_3_(n). The equation is expressed as

11 $$Y(n) = a_{1} (n) + a_{2} (n) + a_{3} (n)$$

**Figure 6:**
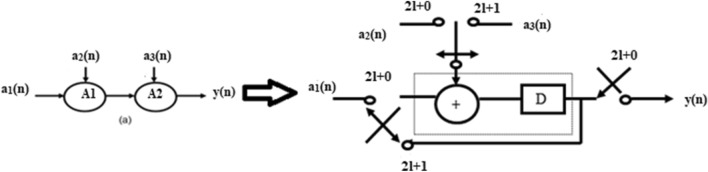
Folded adder.

The original program and folded structure are shown in Fig. 6. During the first clock cycle 2 l + 0, the folded adder performs the operation of A_1_. The left and top switches are closed and produce output a_1_(n) + a_2_(n). The result is temporarily stored in the register. During the second clock cycle 2 l + 1, the folded adder performs the function of A_2_.The input a_3_(n) gets added with result stored in register and produces a_1_(n) + a_2_(n) + a_3_(n).The result y(n) is computed at the next 2 l + 0 instance.

The conventional GLRT block^4^ presented in Fig. 3 consists of two stages and each stage consists of three blocks. The block1 and block2 shown in Fig. 3 is folded using a register minimization technique. Since block2 and block3 are identical in both stages, the folded block2 is replaced by block3 in final GLRT block.

### Folding of GLRT Block 1

**Figure 7:**
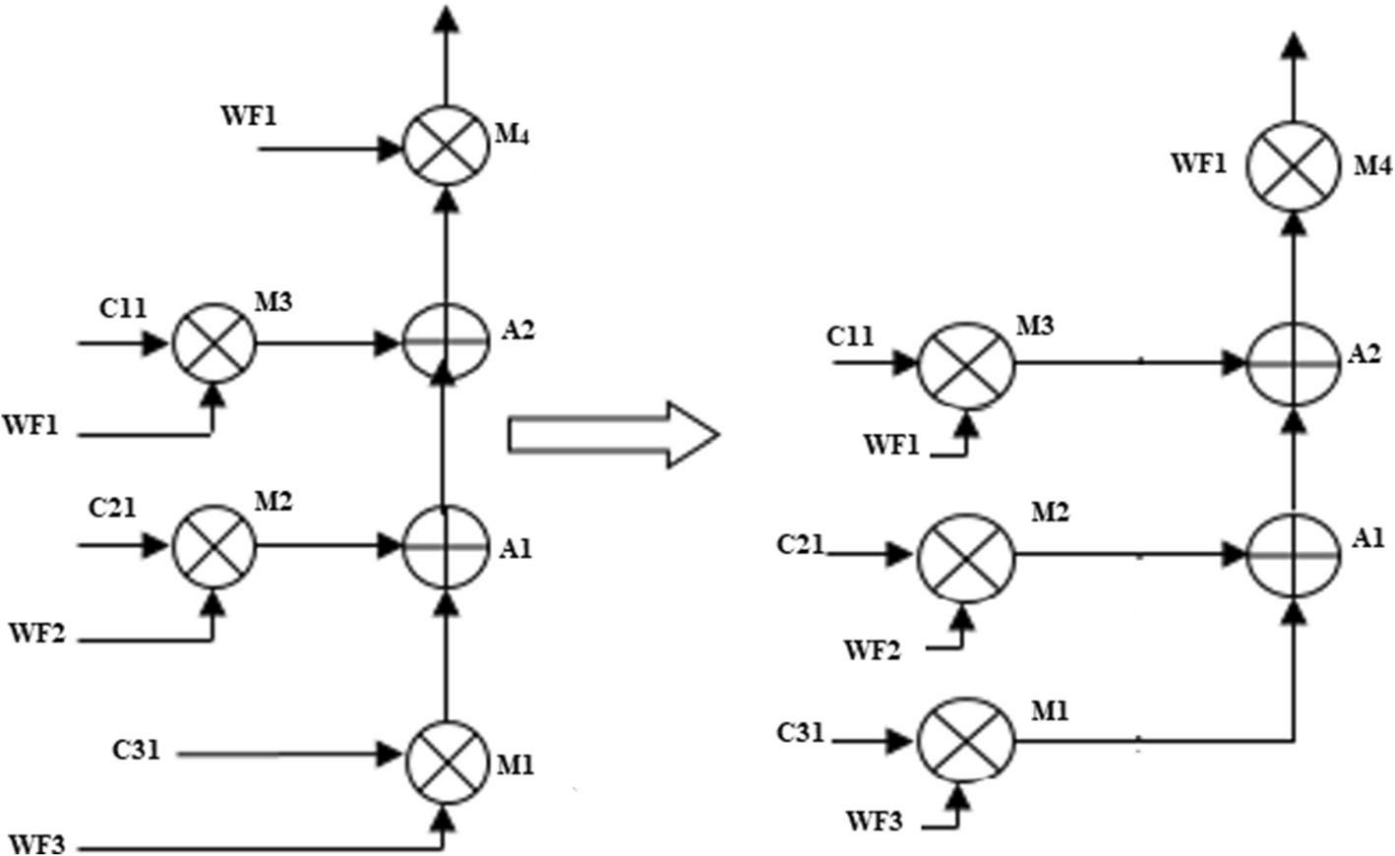
Block1 of GLRT.

GLRT block1with corresponding Data Flow Graph (DFG) is shown in Fig. 7. The folding factor N is defined as operations folded by the same functional units^20^. A Folding set is an ordered set executed by the same functional block. The set consists of N operations and the order of operations is 0 to N-1. The block1 of the GLRT consists of four multipliers and two adders. The four multipliers and adders in block1 are folded as single adder and multiplier. So the folding set is taken as S_M_ for a set of multipliers and set of adders as S_A_. The folding order of a node is time scheduled for a particular operator to perform its execution in hardware. The GLRT is folded with folding order of N = 4 and folding sets are presented as (12)

12 $$S_{A} = \{ A_{1,} A_{2,} \varphi ,\varphi \} , S_{M} = \{ M_{1,} M_{2,} M_{3,} M_{4} \}$$

The folding order N = 4 denotes that the iteration period of folded architecture is 4 units and each functional unit executes 4 operations in hardware. The set S_A_ consists of adders A_1_ and A_2_ which has folding order 1, 2. During time unit 4 l + 0, the folded adder performs the function of A_1_ and at time unit 4 l + 1 performs as A_2_. A null operation (ø) in the folding set S_A_ indicates no operation. The null operations are included to produce an equal number of operations in both adder and multiplier sets. The number of delay elements required in the folded architecture between the source and the destination node is calculated using the folding Eq. (13).The computation time of adder is assumed to be T_A_ = 1u.t and T_M_ = 2u.t^19,20^.

13 $$D_{F} (U \to V) = Nw(e) - P_{u} + v - u$$

Here U and V are the source and destination nodes of a DFG. A w (e) is the delay between the source and destination node. P_U_ indicates the pipelined delay for the source node and v, u indicates the folding order that is time scheduled for that hardware unit to perform its operation. The internal structure of Block1 with five edges in a DFG.

Each multiplier has coefficients C_11_, C_21_, C_31_ and inputs WF1, WF2, WF3. The multipliers are renamed as M_4_, M_3_, M_2_, and M_1_.The adders are named as A_1_ and A_2_. The folding equations are calculated and given in (14)

14 $$\begin{gathered} D_{F} (A_{2} \to M_{4} ) = (4*0) - 1 + 3 - 1 = 1 \hfill \\ D_{F} (A_{1} \to A_{2} ) = (4*0) - 1 + 1 - 0 = 0 \hfill \\ D_{F} (M_{1} \to A_{1} ) = (4*0) - 2 + 0 - 0 = - 2 \hfill \\ D_{F} (M_{2} \to A_{1} ) = (4*0) - 2 + 0 - 1 = - 3 \hfill \\ D_{F} (M_{3} \to A_{2} ) = (4*0) - 2 + 1 - 2 = - 3 \hfill \\ \end{gathered}$$

The folded structures are realizable only when the delays are positive. But the above Eq. (14) have negative delays and unrealizable. So cutset retiming is a transformation technique applied to DSP programs to reduce the clock period. Retiming adds a delay in a critical path and reduces the clock period of the architecture^20^. The cutset retiming add a delay in the path where negative delays exist and makes edges with positive delays^20^.The first step of cutset retiming is introduction of cutset in DFG as shown in Fig. 7. A single cutset produces two different subgraphs G1 and G2. The subgraph G1 has nodes M_3_, M2, M1 and G2 graph has A_1,_ A_2_, M_4_. The second step is adding a delay element in the edges which moves from G1 to G2 and then subtract a delay from edges moving from G2 to G1. In this DFG, there is only feedforward edges and one delay get increased in the corresponding edges from G1 to G2; for example, the edge weight of the nodes M_1_ to A_1_ , M_2_ to A_1_ , M_3_ to A_2_ gets increased with one delay. Therefore, the delay equations are recalculated and then the edges with positive delays are used in the final folded structure. The edges M_1_ to A_1_, M_2_ to A_1_ and M_3_ to A_2_ have negative edges as shown in Eq. (14) and after cutset retiming the edges remain positive. The folding equations derived after cutset retiming are expressed as (Eq. 15)

15 $$\begin{gathered} D_{F} (A_{2} \to M_{4} ) = (4*0) - 1 + 3 - 1 = 1 \hfill \\ D_{F} (A_{1} \to A_{2} ) = (4*0) - 1 + 1 - 0 = 0 \hfill \\ D_{F} (M_{1} \mathop{\longrightarrow}\limits^{D}A_{1} ) = (4*1) - 2 + 0 - 0 = 2 \hfill \\ D_{F} (M_{2} \mathop{\longrightarrow}\limits^{D}A_{1} ) = (4*1) - 2 + 0 - 1 = 1 \hfill \\ D_{F} (M_{3} \mathop{\longrightarrow}\limits^{D}A_{2} ) = (4*1) - 2 + 1 - 2 = 1 \hfill \\ \end{gathered}$$

A folded architecture of block1 requires two delay elements to store intermediate results. So a register minimization technique^21^ is used to implement a folded structure with minimum delays. The steps of the register minimization technique are: construction of life time table, Lifetime chart and data allocation table ^21–20^. Using the folding equations, a life time table is constructed to obtain lifetime of each input variables and as shown in Table 1. The lifetime of each node is calculated using (Eq. 16)

16 $$T_{input} = u + P_{u}$$

where u is the folding order of the source node and P_u_ is the pipelining delay of the source node. The lifetime of each node is calculated using T_input_ and T_output_^20^. The T _output_ is calculated using (Eq. 17)

17 $$T_{output = } u + P_{u} + \max D_{F} (U \to V)$$

**Table 1:** Life time table of Block 1.

Nodes	T_input_	T_input_ → T_output_
A_1_	1	1 → 1
A_2_	2	2 → 3
M_1_	2	2 → 4
M_2_	3	3 → 4
M_3_	4	4 → 5
M_4_	5	5 → 5

**Figure 8:**
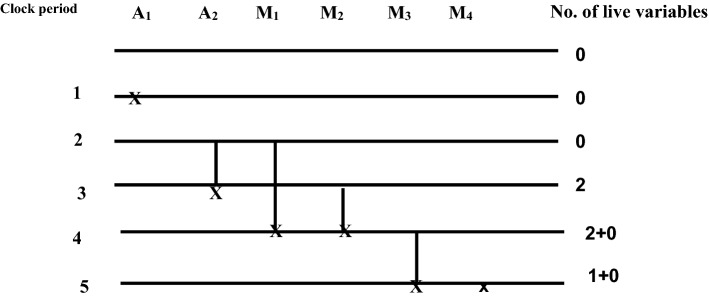
Lifetime chart of Block1.

Using the life time table, a lifetime chart is constructed for the Block1. A graphical representation of a lifetime of variables in a linear fashion is shown in Fig. 8. An input variable is said to be live from production time and till it is consumed. It is assumed to be dead after consumption; for example, A_2_ is assumed to be live during two clock cycles 2, 3. The lifetime chart consists of horizontal lines that represent the clock period and the input variables which are live during each period are shown in vertical lines^20^. The maximum number of live variables during any clock period is determined and this gives a minimum number of registers (delays) used to implement block1.The maximum number of registers needed to implement block1 is max {0, 0, 0, 2, 2, 1} = 2.

The data allocation table^20^ uses two registers R1 and R2. The first step of the data allocation table is allocating each variable that has non zero duration into corresponding registers at the beginning of their lifetime (clock cycle). The input variables are allocated in a forward and backward manner as shown in Table 2. The input variables A_1_, M_4_ has zero duration and are assumed to be dead variables at the time of production.so the variables A_1_, M_4_ are produced and consumed at the same clock cycle1,5. The dead variables reach the output at the corresponding clock cycle.

**Table 2:**
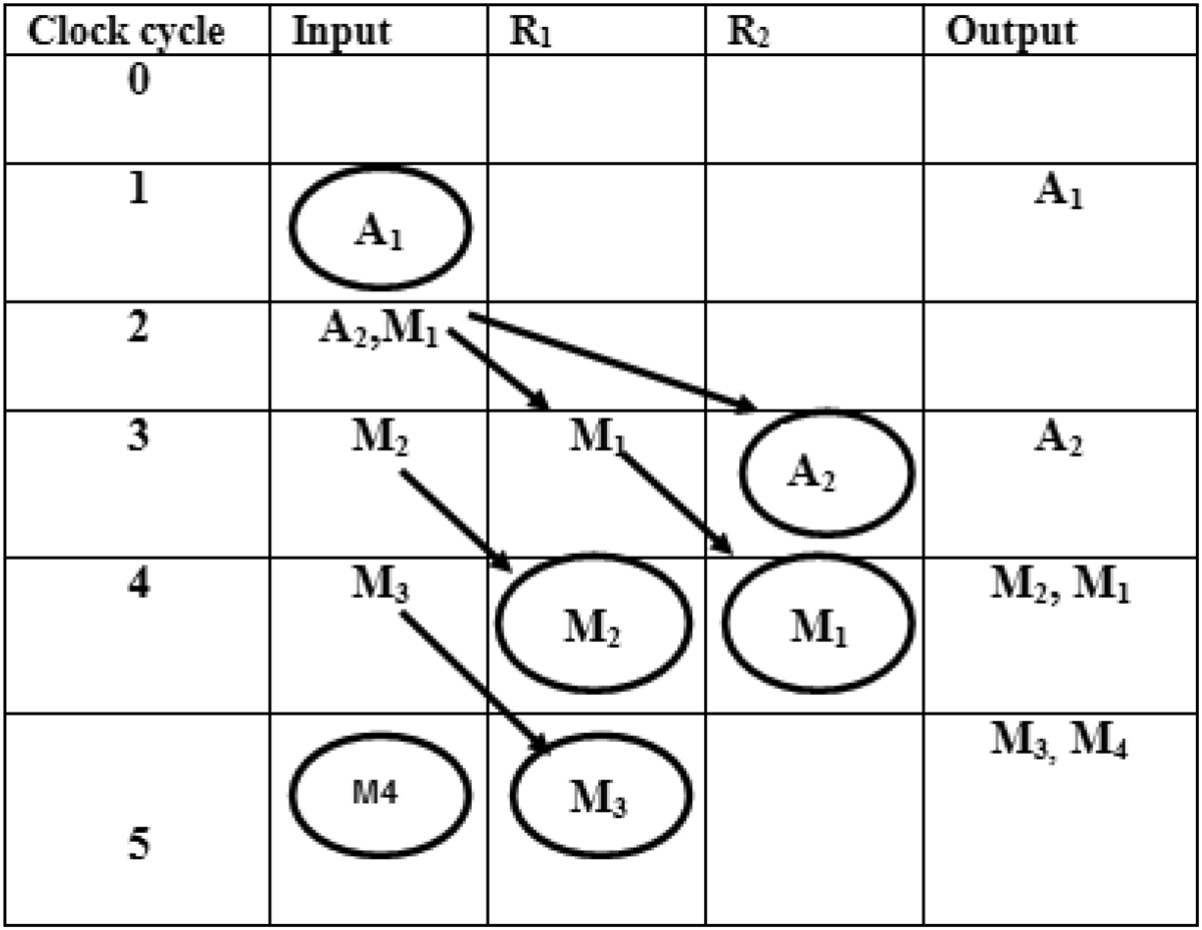
Data allocation table for Block 1.

When multiple variables A_2_, M_1_ has the same lifetime in the beginning, then the allocation is performed based on a lifetime of the variable. Among multiple variables, the input variable with the longest lifetime is allocated to the initial register and other variables to consecutive registers; for example, variable M_1_ has a longer lifetime than A_2_. Accordingly, M_1_ is allocated in register R_1_ at cycle 2 and A_2_ in consecutive register R_2_. Similar allocation is performed for variables M_2_ and M_3_ at corresponding clock cycles. When a variable reaches last register and not dead, a backward allocation is performed. The backward allocation is performed for the remaining life period based on First Come First Served basis^21^.

**Figure 9:**
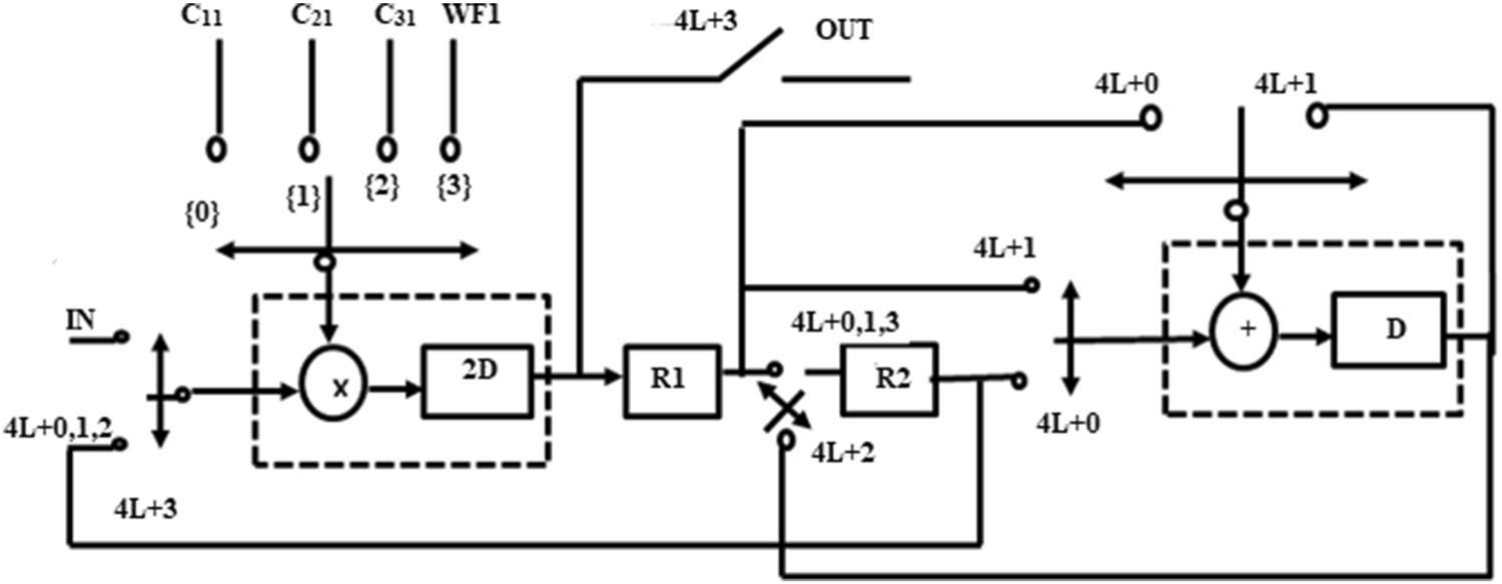
Folded structure of Block1 of GLRT.

The final folded architecture of Block1 with a minimum of 2 registers is shown in Fig. 9. The switch is replaced with 4 × 1 Mux in hardware. The adder has a self-pipelined delay of 1u.t and the multiplier has 2u.t^20^. The set of folding Eq. (10) are used to synthesize the folded architecture with minimum delays. The edge (A_2_–M_4_) has D_f_(A_2_–M_4_) = 1 delay in the folded architecture. There is an edge from adder to a multiplier that passes through R_1_. The input variable A_2_ is stored in register R_1_ and after one delay there is an edge form R_1_ to a multiplier (M_4_) in final folded architecture. This edge is switched into multiplier input at time instance 4L + 3 because the destination node M_4_ has folding order 3. The procedure is repeated for all other edges between adder and multiplier in folded architecture.

**Figure 10:**
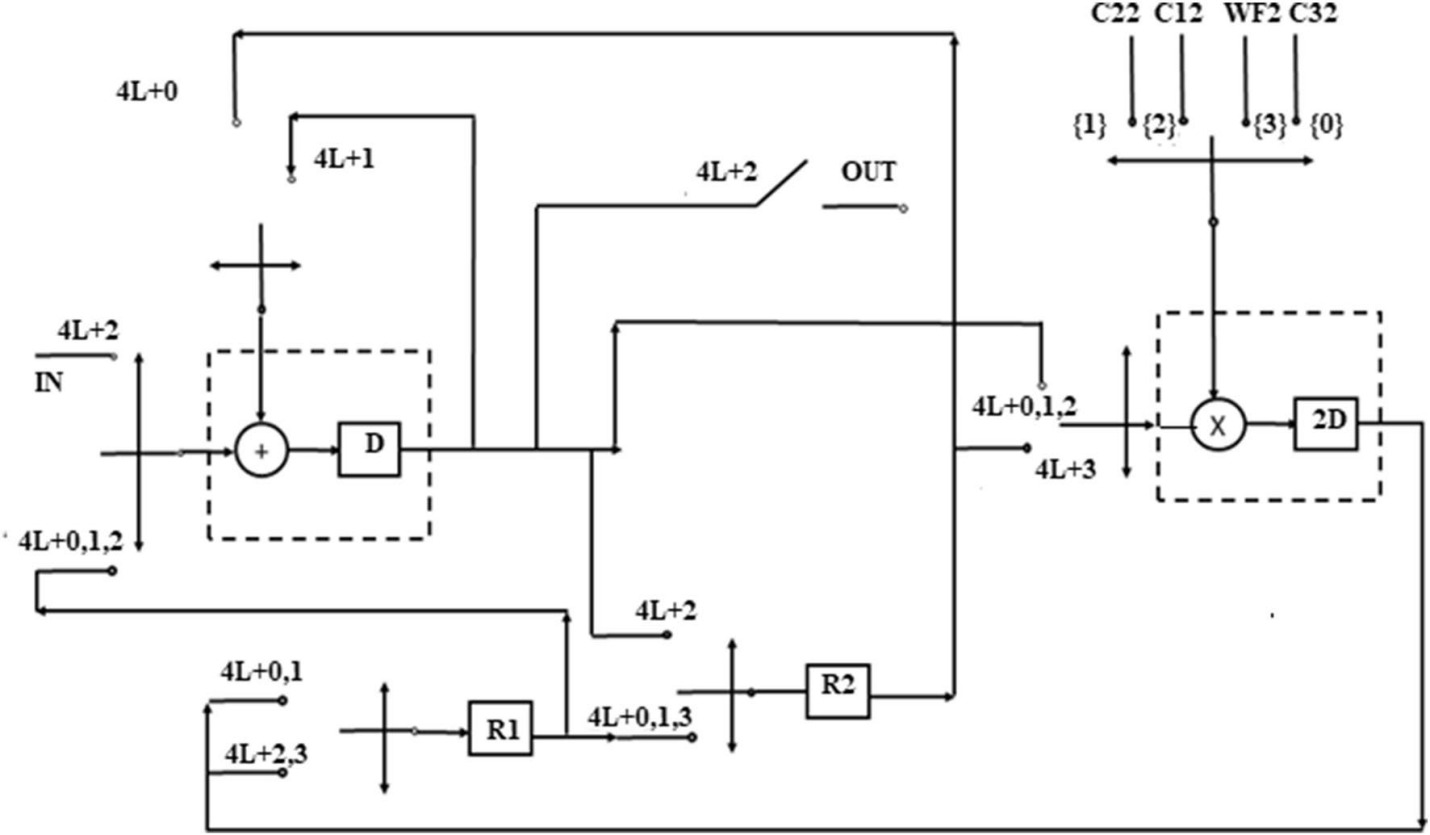
Folded structure of Block2 of GLRT.

Similar to block1, block2 of GLRT structure is folded and final folded architecture is shown in Fig. 10. The block2 of the GLRT consists of four multipliers and three adders. The steps of register minimization technique are applied to block2 and folded with a minimum of two registers.

**Figure 11:**
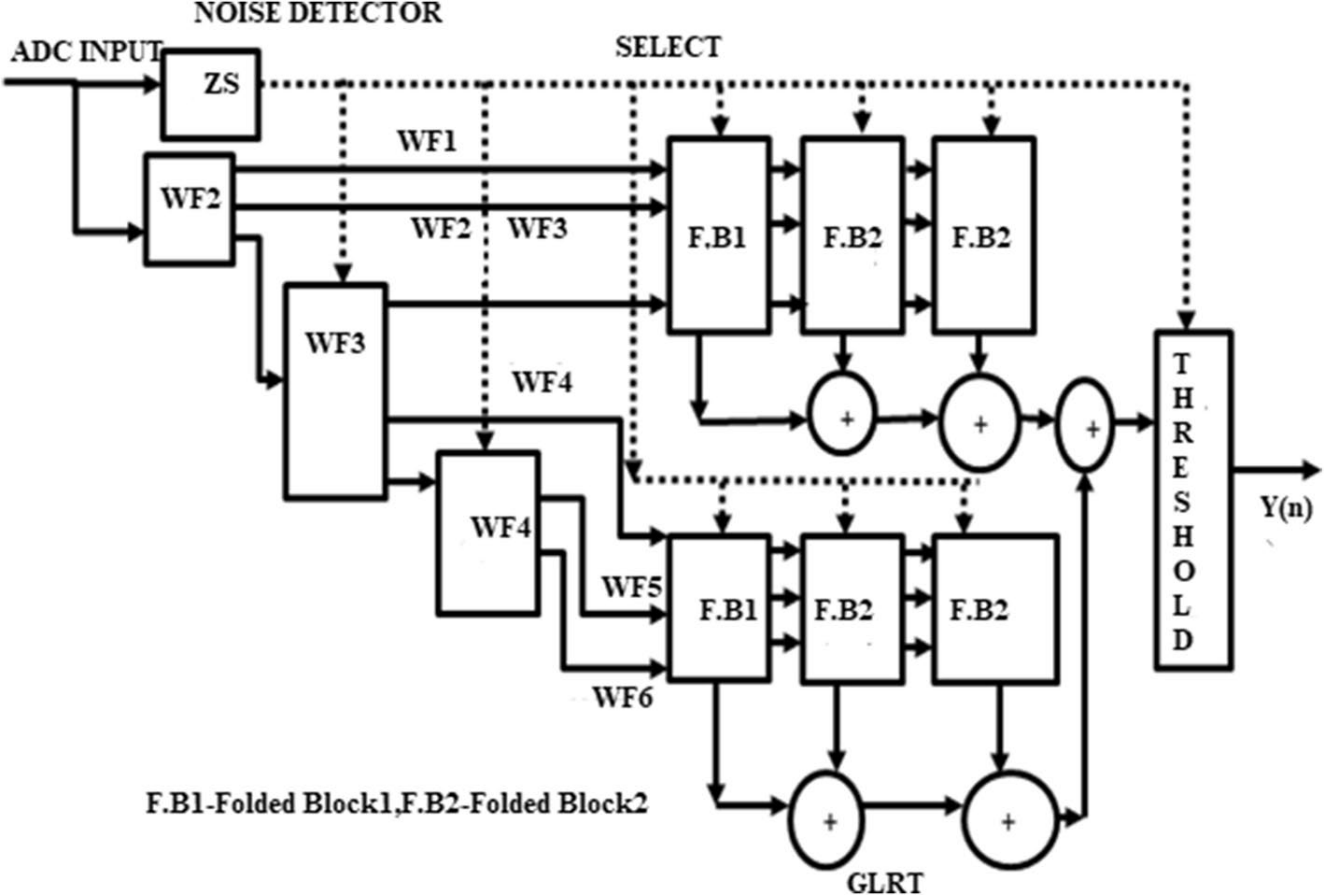
Modified Folded undecimator based ECG detector.

Both the folded blocks are replaced in the entire GLRT structure as shown in Fig. 3. The folded block1 of stage1 is reused in stage2. Similarly, the folded block2 can be replaced in the place of block2 and block3 in both the stages. The modified folded undecimator based ECG detector with folded GLRT blocks are shown in Fig. 11.

## Results and discussion

The modified folded undecimator based ECG detector is implemented and simulated using Xilinx System Generator (XSG) and tested with standard MIT-BIH Arrhythmia database^22^. MIT-BIH Arrhythmia database consists of 48 recordings sampled at 360 Hz and 11 bits resolution with a range of 10 mV^23–25^. The modified architecture is tested with signals of MIT-BIH short time database (10-s), medium database (1-min) and full length database (1-h)^22,26^.The performance of both conventional and modified ECG detectors are evaluated using sensitivity S_e_, Positive prediction P^+^ and Detection Error Rate(DER)^9,23,27^. A beat by beat comparison is performed for signals present in the database and calculated using (18)–(19).

18 $$SensitivityS_{e} = \frac{TP}{{TP + FN}}\%$$

19 $$\begin{array}{*{20}c} {Positive} & {prediction} \\ \end{array} P^{ + } = \frac{TP}{{TP + FP}}\%$$

20 $$DER = FN + FP/Tot.No.QRSComplex$$

where FP- False Positive represents the extra peak reported as QRS candidate and FN- False Negative represents the missed peak when there is a real peak.TP- True Positive represents the number of the correctly detected QRS complex.

### QRS complex detection using MIT-BIH arrhythmia database

**Figure 12:**
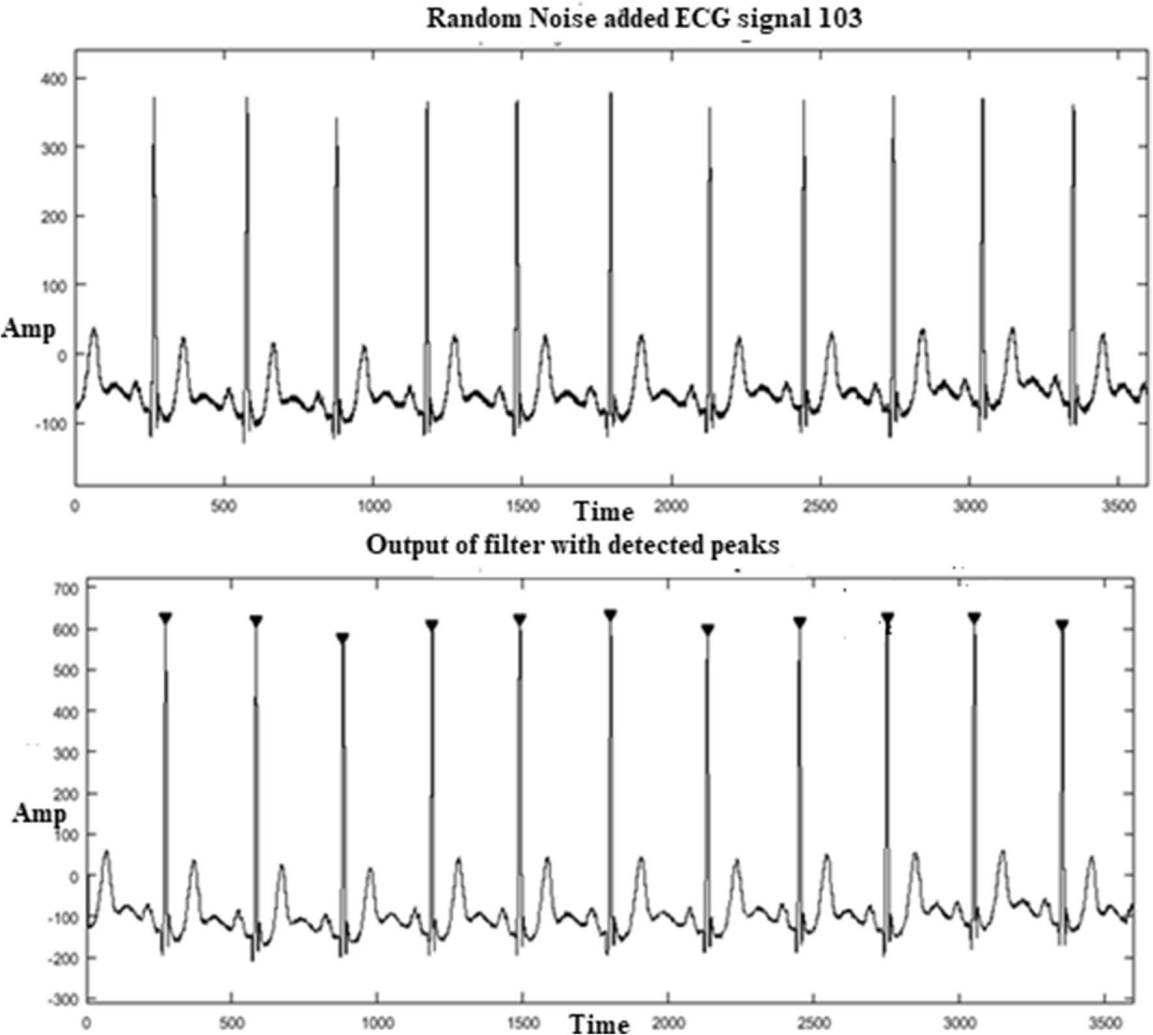
ECG detector output for a short-term input 103.

ECG signal of MIT-BIH arrhythmia is used to evaluate the modified ECG detector. Three different datasets are chosen. The database includes short term database of 10 s, medium data of one-minute and full length data of 1-h duration. The performance parameter is estimated for all the recordings of short term database and is tabulated in Table 5. The output of the modified detector for MIT-BIH arrhythmia short term database is shown in Fig. 12. MIT database for a sample Record 103 is added with random noise and given as input for a modified ECG detector.

The performance of the modified ECG detector using short term ECG recordings of MIT-BIH arrhythmia for 48 samples is summarized in Table 3. The modified structure achieves a sensitivity of 99.31% and predictivity of 99.67% and DER of 0.001 with short term recordings. The signals 108.mat and 214.mat from a MIT-BIH arrhythmia has maximum noise^9^. The modified folded detector produces 100% on the 214 signal. But the signal 108.mat has 91.7% predictivity and DER of 0.090. The modified detector outperforms and produces 100% for the other signals present in the short-term database.

**Table 3:** Performance of modified folded ECG detector with short term database.

Record no	Total no. of beats	TP (beats)	FN (beats)	FP (beats)	S_e_ (%)	P^+^ (%)	DER
**Performance of modified folded ECG detector (folded GLRT-adder, multiplier)**							
100	13	13	0	0	100	100	0
101	11	11	0	0	100	100	0
102	12	12	0	0	100	100	0
103	11	11	0	0	100	100	0
104	13	12	1	0	92.3	100	0.076
105	14	14	0	1	100	93	0.071
106	10	10	0	0	100	100	0
107	12	12	0	0	100	100	0
108	11	11	0	1	100	91.7	0.090
109	16	16	0	0	100	100	0
111	12	12	0	0	100	100	0
112	14	14	0	0	100	100	0
113	9	9	0	0	100	100	0
114	10	10	0	0	100	100	0
115	10	10	0	0	100	100	0
116	14	14	0	0	100	100	0
117	9	9	0	0	100	100	0
118	12	11	1	0	91.66	100	0.083
119	10	10	0	0	100	100	0
121	10	10	0	0	100	100	0
122	15	15	0	0	100	100	0
123	9	9	0	0	100	100	0
124	8	8	0	0	100	100	0
200	15	15	0	0	100	100	0
201	14	14	0	0	100	100	0
202	7	7	0	0	100	100	0
205	15	15	0	0	100	100	0
207	10	10	0	0	100	100	0
208	13	13	0	0	100	100	0
209	15	15	0	0	100	100	0
210	16	16	0	0	100	100	0
212	15	15	0	0	100	100	0
213	18	18	0	0	100	100	0
214	13	13	0	0	100	100	0
215	18	18	0	0	100	100	0
217	12	11	1	0	91.66	100	0.083
219	13	13	0	0	100	100	0
220	12	11	1	0	92.3	100	0.076
221	13	13	0	0	100	100	0
222	13	13	0	0	100	100	0
223	13	13	0	0	100	100	0
228	12	12	0	0	100	100	0
230	14	14	0	0	100	100	0
231	10	10	0	0	100	100	0
232	9	9	0	0	100	100	0
233	17	17	0	0	100	100	0
234	15	15	0	0	100	100	0
Mean	587	583	4	2	99.31	99.67	0.001

**Figure 13:**
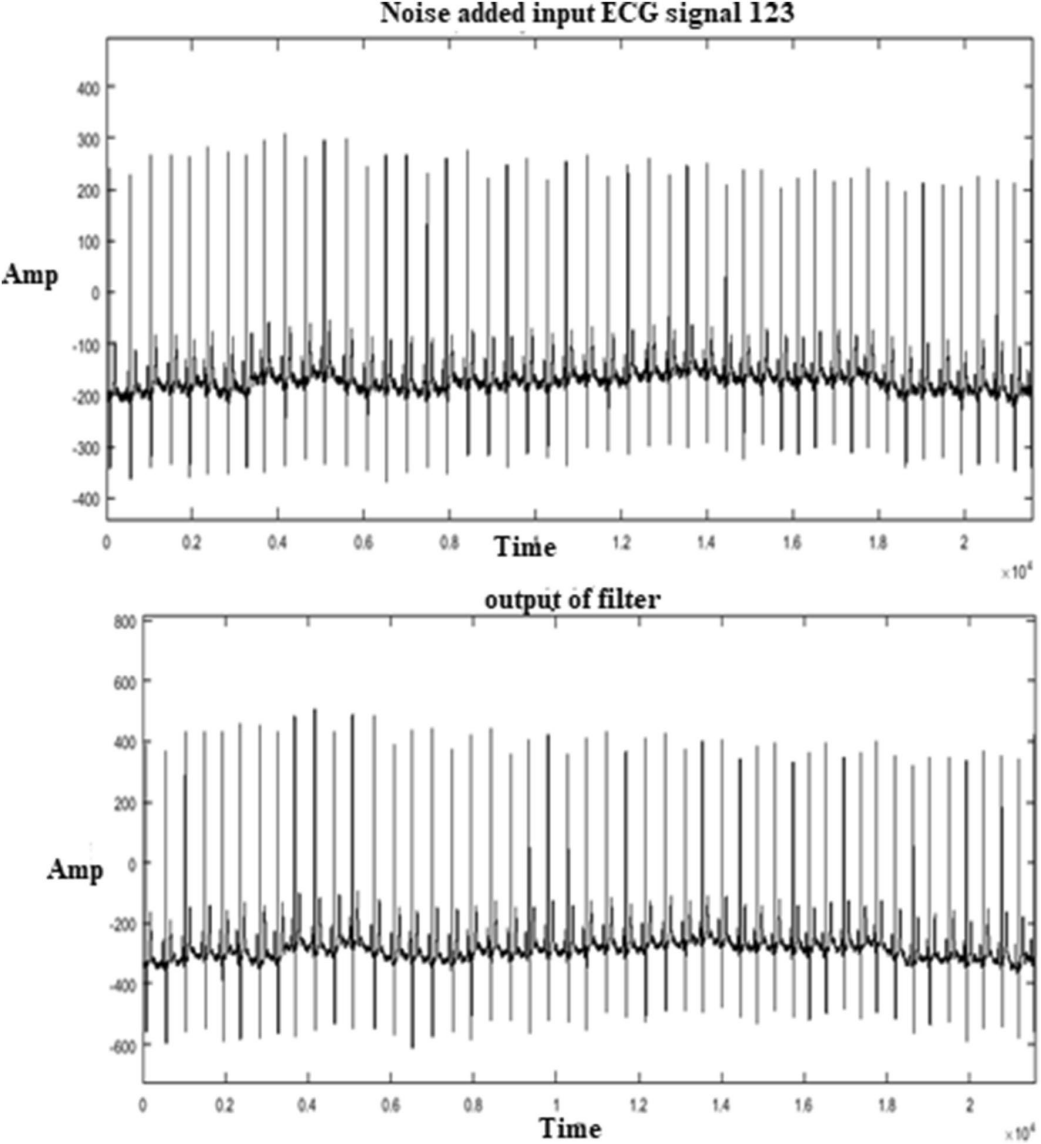
ECG detector output for a long term input 123.

The output of the modified detector for MIT-BIH arrhythmia long term database is shown in Fig. 13. MIT database for Record 123 is added with random noise and given as input for a modified Detector.

The performance comparison of the modified ECG detector for MIT-BIH arrhythmia full length (1 h) database is shown in Table 4. The modified structure achieves a sensitivity of 99.95% and predictivity of 99.97% and DER of 0.061 with full-length database.

**Table 4:** Performance of modified folded ECG detector with 1-h database.

Record no	Total no. of beats	TP (beats)	FN (beats)	FP (beats)	S_e_ (%)	P^+^ (%)	DER
**Performance of modified folded ECG detector (folded GLRT-adder, multiplier)**							
100	2273	2273	0	0	100	100	0
101	1865	1864	1	0	99.95	100	0.053
102	2187	2186	1	0	99.95	100	0.046
103	2084	2084	0	0	100	100	0
104	2229	2222	5	3	99.78	99.86	0.35
105	2572	2568	4	2	99.84	99.92	0.23
106	2027	2025	2	2	99.90	99.90	0.198
107	2137	2135	2	0	99.90	100	0.093
108	1763	1761	2	1	99.77	99.95	0.170
109	2532	2532	0	0	100	100	0
111	2124	2122	2	0	99.91	100	0
112	2539	2539	0	0	100	100	0
113	1795	1795	0	0	100	100	0
114	1879	1878	1	0	99.94	100	0.053
115	1953	1953	0	0	100	100	0
116	2412	2412	0	0	100	100	0
117	1535	1535	0	0	100	100	0
118	2288	2288	0	2	100	99.91	0.08
119	1987	1987	0	0	100	100	0
121	1863	1863	0	0	100	100	0
122	2476	2476	0	0	100	100	0
123	1518	1517	0	0	100	100	0
124	1619	1619	1	0	99.93	100	0.066
202	2136	2136	0	0	100	100	0
203	2980	2976	3	2	99.89	99.93	0.16
209	3005	3005	0	0	100	100	0
215	3363	3363	0	0	100	100	0
217	2208	2203	5	2	99.77	99.90	0.31
220	2048	2046	2	0	99.91	100	0.09
223	2605	2605	0	1	100	99.96	0.038
230	2256	2255	1	0	99.96	100	0.044
234	2753	2753	0	0	100	100	0
Mean	71,011	70,976	32	15	99.95%	99.97%	0.061

### Performance comparison of ECG detectors

The summary of ECG detection performance for different databases of MIT-BIH arrhythmia (47 recordings) is tabulated in Table 5. It is clear that the modified ECG detector achieves S_e_ and P^+^ similar to existing ones and the DER is lesser than the other structures. Similarly, the performance of long-term database is compared with different structures.

**Table 5:** Performance of ECG detectors on short and medium-length database.

	Duration	Total peaks	S_e_ (%)	P^+^ (%)	DER
Modified folded	10 s	587	99.31	99.67	0.001
	1 min	3144	99.65	99.55	0.007
BWT^9^	10 s	587	99.31	99.65	1.02
	1 min	3144	99.65	99.65	0.006

A comparison of modified folded architecture with the existing works is summarized in Table 6. The modified detector produces better performance than the other structures.

**Table 6:** Performance of ECG detectors on 1-h database.

	S_e_ (%)	P^+^ (%)	DER
Modified folded	99.95%	99.97%	0.061
Dyadic WT^4^	99.91%	99.17%	0.952
Dyadic WT^25^	99.91%	99.63%	0.260

### Hardware comparison of ECG detectors

Hardware comparison of Modified ECG detector with different WFB realization structures is tabulated in Table 7. The hardware complexity is estimated based on the requirement of LPF, HPF, Adders, Multipliers and delay elements present in Wavelet Filter Bank. It is observed that a modified folded structure requires few hardware than the other structures. In this work, Folding transformations are applied only to the GLRT block. When a folding technique is applied to the WFB, the hardware complexity of WFB can be reduced when compared with the other existing works. A folded LPF can be implemented with one adder, multiplier, and few delay elements. The combined folded WFB with GLRT is hardware efficient.

**Table 7:** Hardware comparison of WFB with existing structures.

	LPF	HPF	Adders	Multiplier	Delays
Modified (folded)	3	4	13	10	13
Dyadic WT^4^	3	4	16	13	13
Dyadic WT^25^	3	4	16	13	13
Bhavotash^28^	3	4	16	13	13
Wavelet^10^	3	4	16	13	13

Compared to the existing techniques, the novelty of the modified work uses a Folding transformation in GLRT block. Hardware requirements are compared with conventional undecimator detector which uses GLRT block. A Folded GLRT based structure of a modified detector uses a single time-multiplexed adder, multiplier and two delay elements for a block. Two stages of GLRT need 6 adders and 6 multipliers. The final adder to produce T(n) makes some adders required as 7. Two delay elements (R1, R2) are used to store the intermediate results in each block of the final folded GLRT block. It is evident from Table 8, that Modified undecimator based detector has area savings of 59%interms of adders and 75% in terms of multipliers. The trade-off occurs between area and delay elements in the folded GLRT block.

**Table 8:** Comparison of GLRT based ECG detector.

	Wavelet filter bank					GLRT block		
	LPF	HPF	Adder	Multiplier	Delays	Adder	Multiplier	Delays
Dyadic WT^4^	3	4	16	13	13	17	24	–
Modified folded	3	4	13	10	13	7	6	12
Area savings			18.75%	23%		59%	75%	

The Folded GLRT requires 7 adders and 6 multipliers. The area needed to realize adders are 2030 µm^2^ and power consumed is 714 µW. Similarly, multipliers need 12,951.36 µm^2^ and power consumed are 2611.86 µW. The area and power consumption results are shown in Table 9 and it is comparatively lower than the conventional GLRT structure.

**Table 9:** Comparison of GLRT based ECG detector.

Methods	Adders	Area required for adders (290 µm^2^ per adder)^29^	Power required for adders (102 µW per adder)^29^	Multipliers	Area required for multipliers (2158.56 µm^2^ per multiplier)^30^	Power required for multipliers (435.31 µW per multiplier)^30^
Dyadic WT^4^	17	4930 µm^2^	1734 µW	24	51,805.44 µm^2^	10,447.44 µW
Modified folded	7	2030 µm^2^	714 µW	6	12,951.36 µm^2^	2611.86 µW
Area savings		59%			75%	

### Synthesis results of folded GLRT

**Figure 14:**
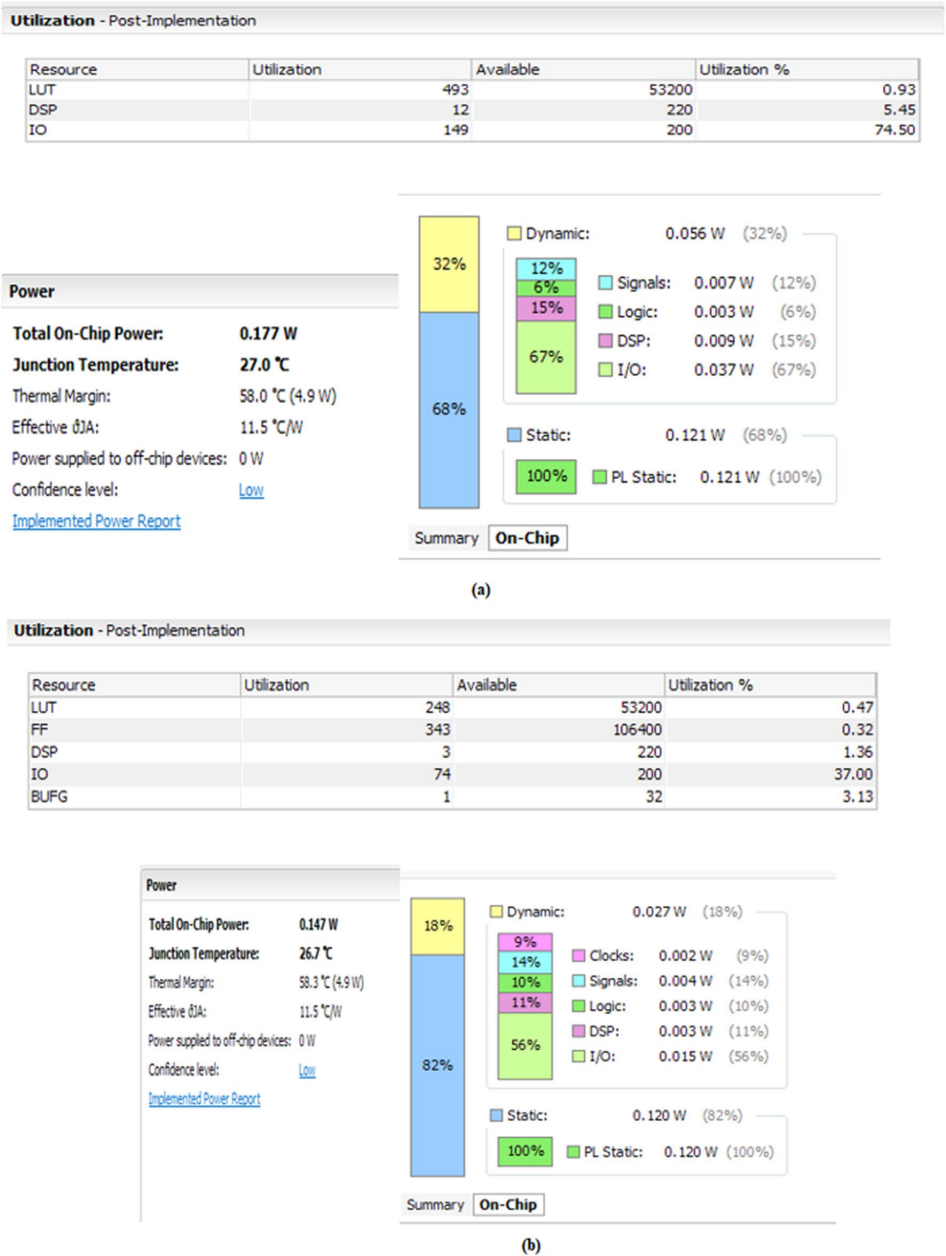
Synthesized results for folded GLRT block (**a**), conventional block (**b**) folded GLRT block.

The novelty of the paper focuses on folding which is a high-level transformation technique effectively mapped with FPGA. The conventional GLRT and folded GLRT block are synthesized for the target FPGA device Zed board XC7Z010CLG484-1^31^. The hardware mapping with post-implementation results is summarized in Table 10. Resource utilization is shown in terms of the number of DSP blocks, the number of LUTs and IOs required. The static, dynamic and on-chip power are estimated and shown as synthesis result in Fig. 14a,b.

**Table 10:** Hardware utilization of GLRT and folded GLRT block.

	GLRT				
	LUT	DSP	IO	Static power (%)	Dynamic power (%)
Dyadic WT^4^	493	12	149	68	32
Modified folded	248	3	74	82	18
Area savings of folded GLRT (%)	49	75	50	–	–

The future scope of the work is to apply high-level folding transformations in wavelet filter banks that are used in pacemakers. Due to high system complexity, most of the existing ECG monitoring systems perform detection and data compression independently. A long-term ECG monitoring requires a large memory to store and transmit raw ECG data. Therefore, data compression makes an efficient transmission with large bandwidth^23,32^. Using the high-level transformations, a combined system can be developed which performs detection and data compression with minimal hardware. Low power consumption and speed also can be achieved with high accuracy rate. The applications of the folded architectures can be extended in the other health care monitoring devices.

## Conclusion

A new area-efficient folded wavelet-based ECG Detector has been proposed. The GLRT block of the Modified ECG detector is designed using VLSI Signal processing algorithms like Folding transformation and cutset retiming. The folded structure is adopted with minimal hardware implementation on a silicon chip. The modified folded ECG detector is tested using a real-time ECG signals obtained from the standard MIT-BIH Arrhythmia database. The modified ECG detector has area savings of 59% and multiplier reduction 75% than the conventional detector. When DSP algorithms are implemented at a particular architectural level, the folded transformation enhances the sustainability of the architecture with less hardware. This paper highlights that a folded architecture can be implemented using less area and low power. A Xilinx FPGA realization of the folded structure highlights that the 49% reduction in terms of required DSP blocks and achieves 43% of dynamic power reduction which is comparatively less than the conventional structure.
